# Supplementary material for: Immune selection determines tumor antigenicity and influences response to checkpoint inhibitors
Source: Nat Genet. 2023 Mar 9;55(3):451–60. doi: 10.1038/s41588-023-01313-1 (PMC10011129; doi:10.1038/s41588-023-01313-1)
Supplement: Supplementary file 1 — Supplementary Figs. 1–16. [file 41588_2023_1313_MOESM1_ESM.pdf]

nature genetics

Article https://doi.org/10.1038/s41588-023-01313-1

Immune selection determines tumor antigenicity and influences response to checkpoint inhibitors

In the format provided by the authors and unedited

Immune selection determines tumor immunogenicity and influences response to checkpoint inhibitors

Supplementary Figures

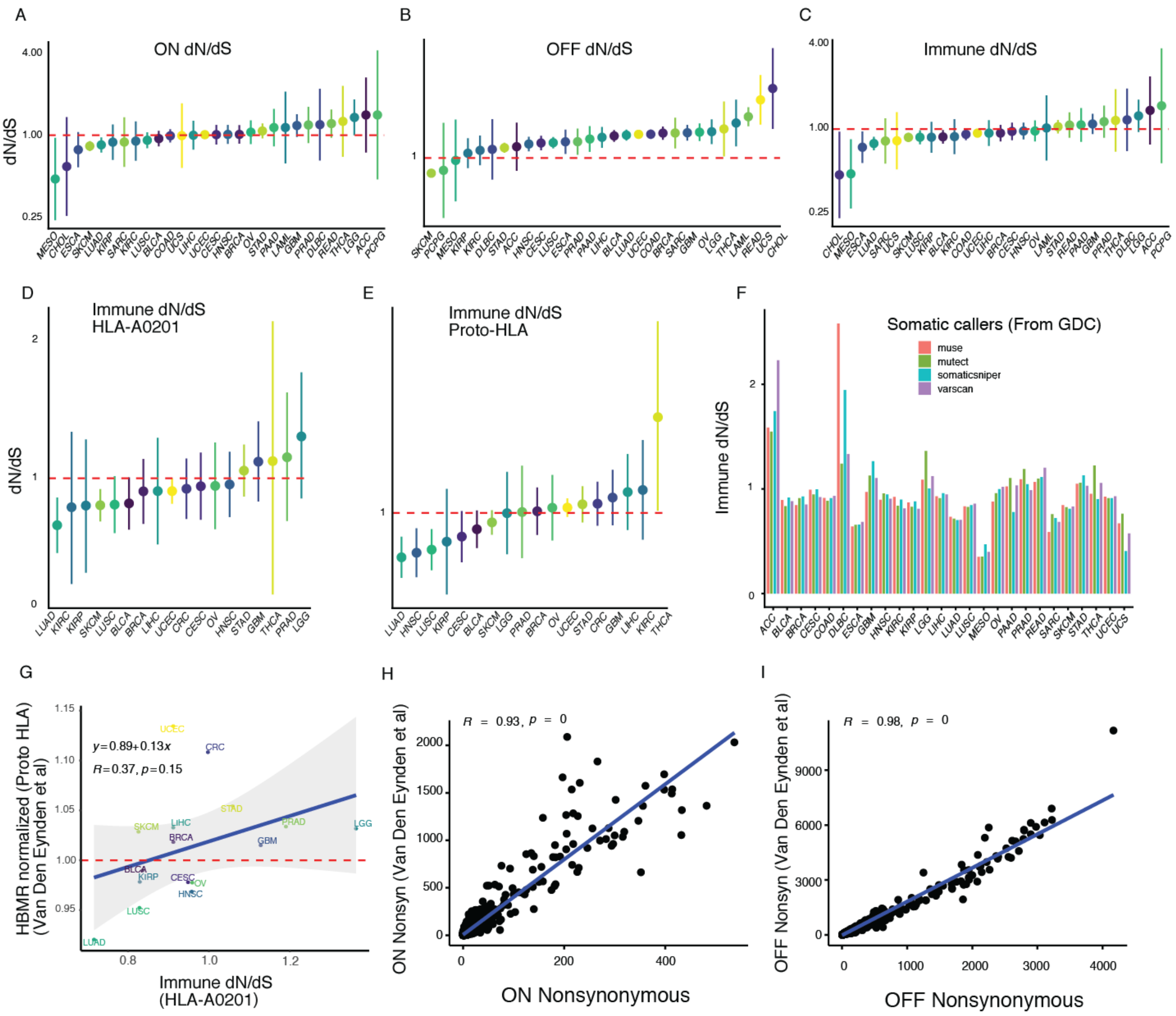

Supplementary Figure 1. Cohort values for 29 TCGA tumour types calculated using the HLA-A0201 immunopeptidome for A) ON-target dN/dS B) OFF-target dN/dS and C) Immune dN/dS. Immune dN/dS for D) HLA-A0201 and E) Proto-HLA immunopeptidomes using randomized mutations. F) Immune dN/dS for TCGA tumour types using different somatic calls from four different callers. Number of samples for each tumor type is described in supplementary table 1. G) Reported point estimates for HBMR Proto-HLA versus SOPRANO immune dN/dS HLA-A0201. Nonsynonymous mutations H) inside and I) outside the HLA-A0201 immunopeptidome. Error bars indicate 95% confidence interval to the point estimate obtained with SOPRANO. P values and correlation coefficients were calculated using Pearson's correlation (two-sided t-test). Error bands indicate the 95% confidence interval.

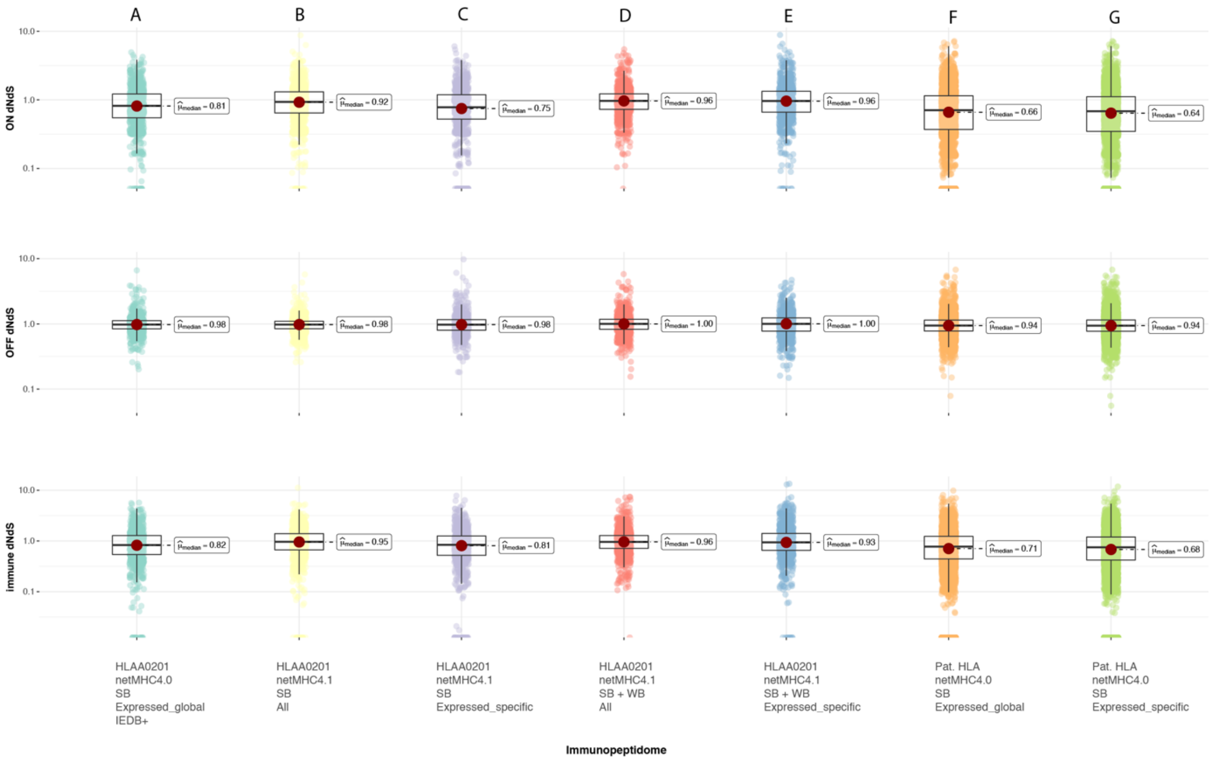

Supplementary Figure 2. SOPRANO ON, OFF and immune dN/dS for different immunopeptidomes. A) Original immunopeptidome used in this study based on predicted strong binders to HLA-A0201 using netMHCpan4.0. Transcripts were filtered based on global expression and similarity to IEDB T-cell positive epitope assays (n=816). B) Immunopeptidome based on predicted strong binders to HLA-A0201 using netMHCpan4.1 (n=861). C) Transcripts from Immunopeptidome B filtered by patient specific expression (n=719). D) Transcripts from Immunopeptidome B including predicted weak binders (n=956). E) Transcripts from Immunopeptidome D filtered by patient specific expression (n=868). F) Original immunopeptidome used in this study based on predicted strong binders to patient specific HLA alleles using netMHCpan4.0 filtered by global expression (n=3828). G) Transcripts from immunopeptidome F filtered by patient specific expression (n=4272). Boxplots represent the median, 25th percentile, and 75th percentile, and whiskers correspond to 1.5 times the interquartile range.

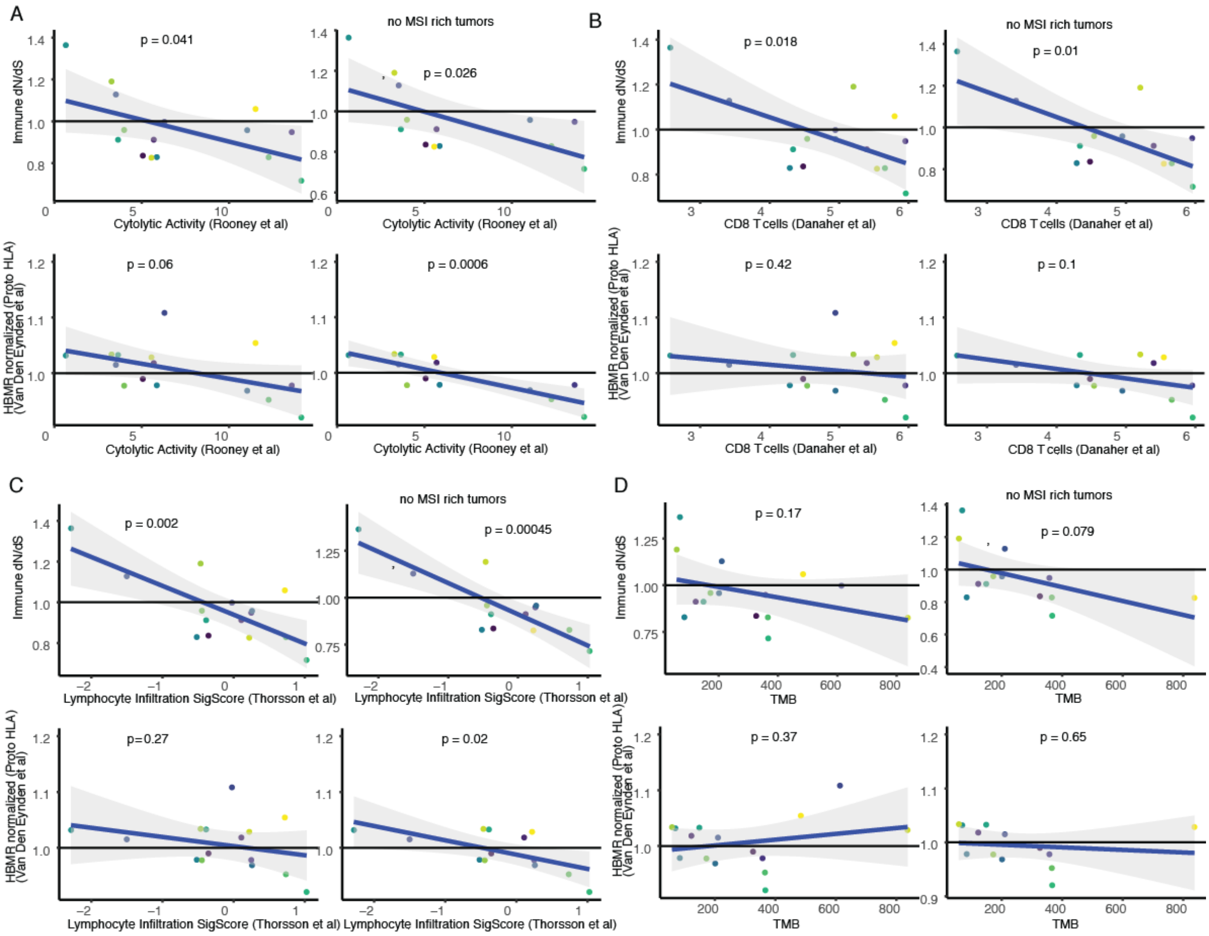

Supplementary Figure 3. Linear regression models for immune dN/dS calculated using SOPRANO and HBMR values versus A) cytolytic activity measurements from Rooney et al 2015, B) CD8 T cells from Danaher et al 2017, C) Lymphocyte infiltration score from Thorsson et al 2018 and D) Tumor mutation burden. For each figure, we present the results including (top left) or excluding (top right) three tumour types with high frequency of escape mechanisms (labelled as "no MSI-rich tumours"): colorectal (CRC), Stomach and Uterine cancer (STAD and UCEC). Each color is the same tumour as described in figure 2 in the main manuscript. P values and correlation coefficients were calculated using Pearson's correlation (two-sided t-test). Error bands indicate the 95% confidence interval.

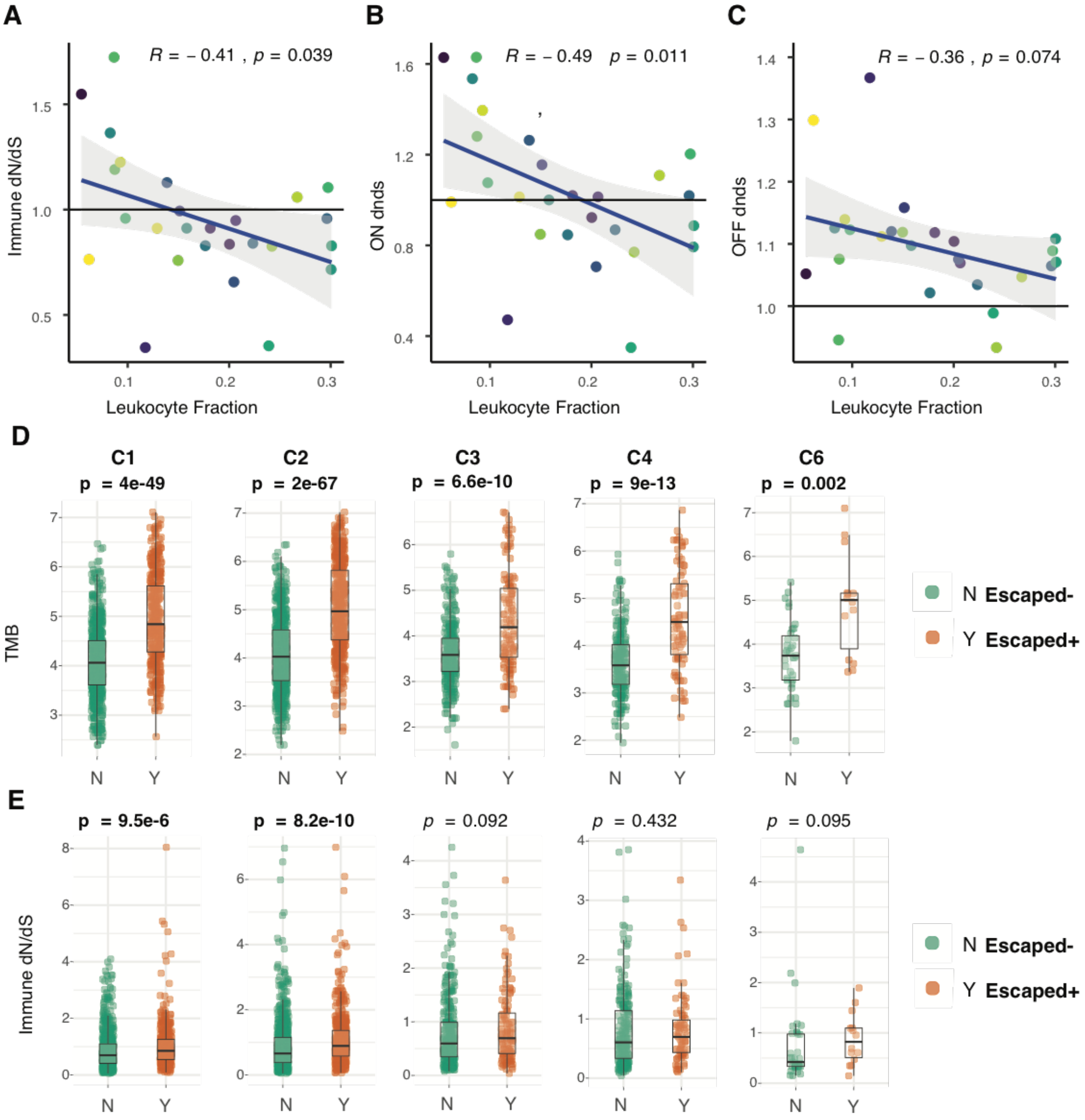

Supplementary Figure 4. SOPRANO A) Immune B) ON-target and C) OFF-target dN/dS values versus leukocyte fraction measurements from Thorsson et al for 33 tumor cohorts. P values and correlation coefficients were calculated using Pearson's correlation (two-sided t-test). Individual estimates of D) tumor mutation burden (TMB) and E) Immune dN/dS from TCGA separated by escape status. Patients were classified into different categories according to Thorsson et al (C1-C6, n=3672). Boxplots represent the median, 25th percentile, and 75th percentile, and whiskers correspond to 1.5 times the interquartile range. P-values reported from a non-parametric two-sided Mann-Whitney U test after multiple test correction using holm method.

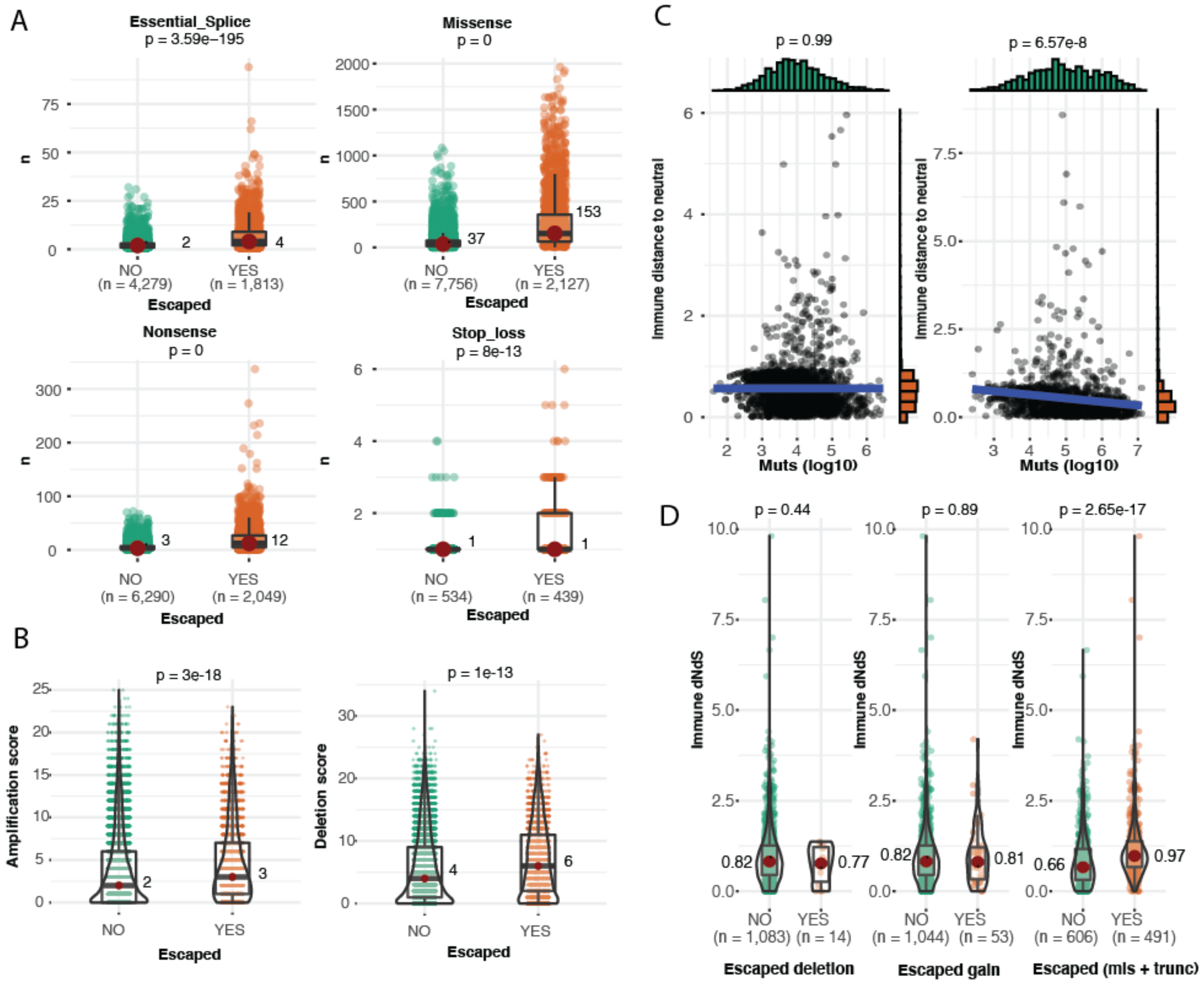

Supplementary Figure 5.A) Number of splice, missense, nonsense, and stop loss mutations in escaped- and escaped+ tumors (Number of individuals with at least one event were analysed). B) Comparison of amplification and deletion scores obtained from Taylor et al 2018 In escaped- (n=7435) versus escaped+ tumors (n=2009). C) Absolute immune distance to neutrality for escaped- (left) and escaped+ (right) tumors (n=3672). D) Immune dN/dS when classifying a subset of tumors as escaped using deletions, gains or missense/truncating mutations in the escape genes defined in the manuscript (n=1097). P values and correlation coefficients were calculated using Pearson's correlation (two-sided t-test). For group comparison, boxplots represent the median, 25th percentile, and 75th percentile, and whiskers correspond to 1.5 times the interquartile range. P-values reported from a non-parametric two-sided Mann-Whitney U test after multiple test correction using holm method.

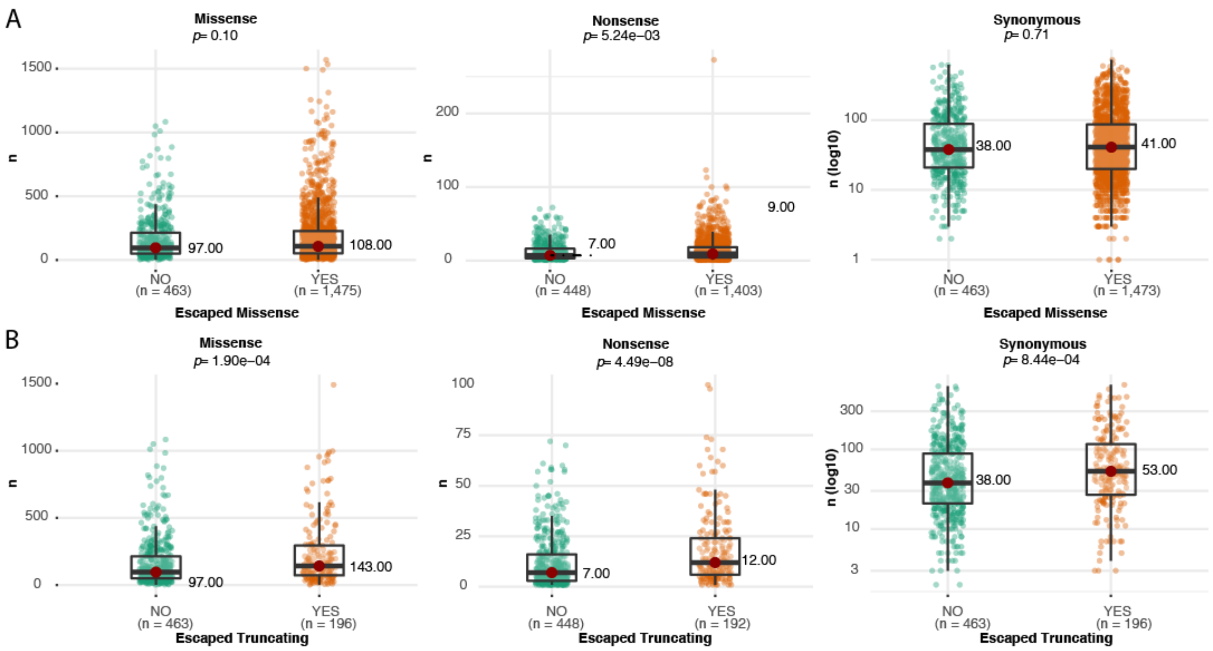

Supplementary Figure 6. Missense, nonsense, and synonymous mutation burden comparison between groups with a A) missense or a B) truncating mutation in an escape gene (YES) and a synonymous mutation (not a missense/truncating) in an escape gene (NO). By using synonymous mutations in escape genes, we control for the mutation bias resulting from selecting mutated genes. Boxplots represent the median, 25th percentile, and 75th percentile, and whiskers correspond to 1.5 times the interquartile range. P-values reported from a non-parametric two-sided Mann-Whitney U test after multiple test correction using holm method.

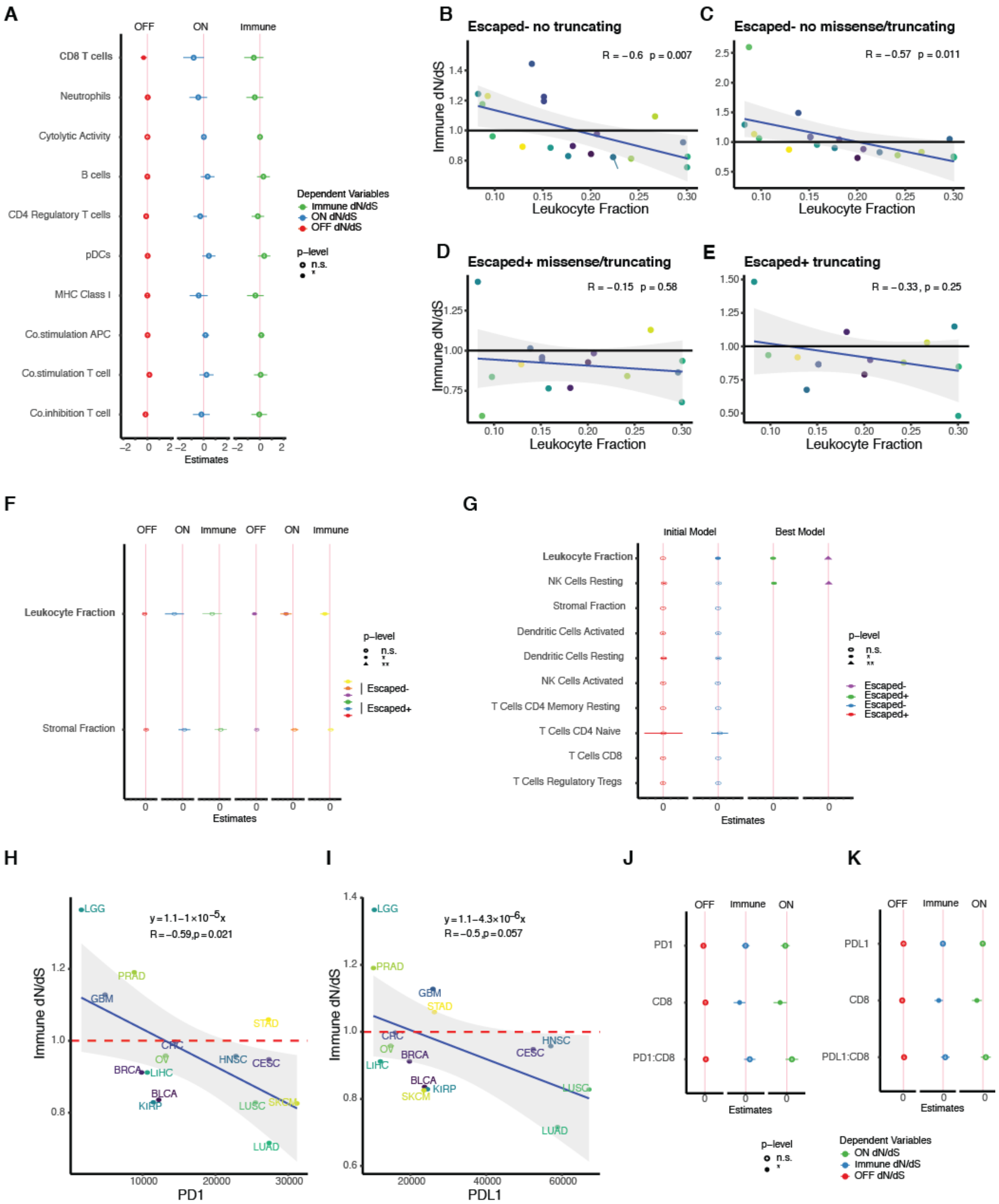

*Supplementary Figure 7. A) Multivariate regression modelling of multiple immune cell metrics against cohort OFF, ON and immune dN/dS. Cohort Immune dN/dS versus Leukocyte fraction when removing patients with B) a truncating mutation or C) a missense or truncating mutation in an escape gene. Cohort Immune dN/dS versus Leukocyte fraction when only including patients with D) a truncating/missense mutation or E) a truncating mutation in an escape gene. F) Multivariate analysis for leukocyte and stromal fraction against OFF, ON and immune dN/dS for escaped- and escaped+ tumors G) Initial and best model for multiple cell metrics against immune dN/dS. Immune dN/dS versus expression (FPKM) levels of H) PD1 and I) PDL1 genes. Multivariate regression model for J) PD1 or K) PDL1, CD8 T cells and their interaction with OFF, ON and immune dN/dS. P values and correlation coefficients were calculated using Pearson's correlation (two-sided t-test). For variable comparison (A,F,G,J,K), significance codes were 0 '***' 0.001 '**' 0.01 '*' 0.05 '.'. Model selection was performed using Akaike information criterion (AIC).*

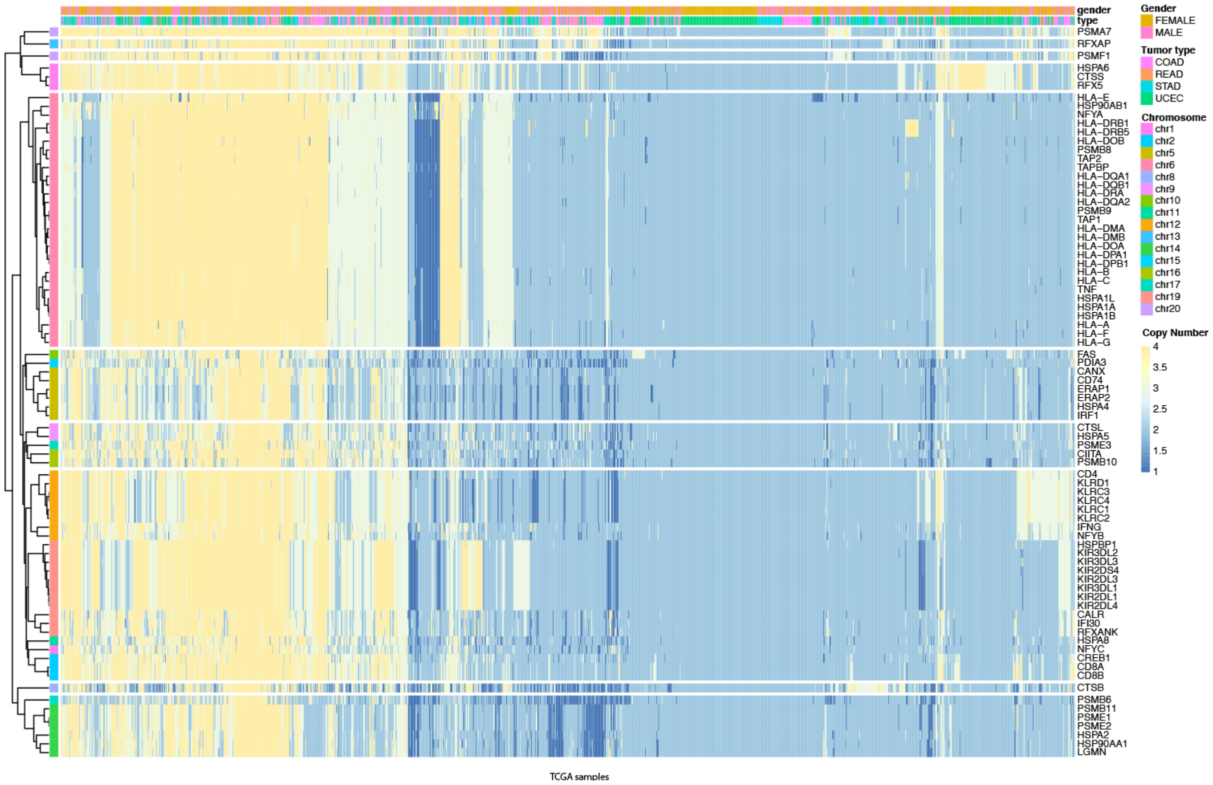

Supplementary Figure 8. Copy number profile for escape genes in 1097 individuals from CRC, STAD, and UCEC tumor types. The copy number profile of HLA genes located on chromosome 6 are clustered together suggesting a strong functional linkage.

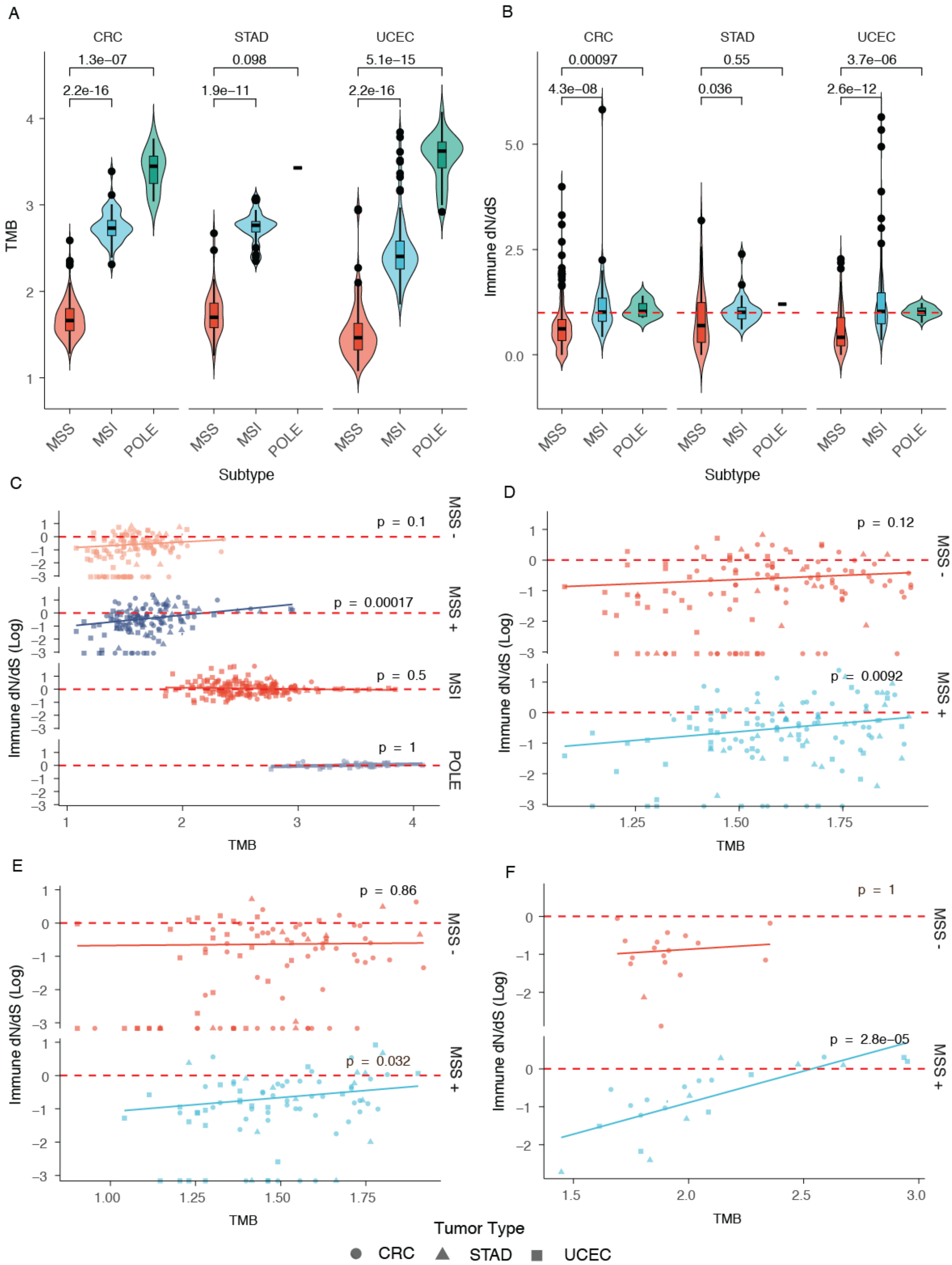

Supplementary Figure 9. A) Tumor mutation burden (TMB) and B) immune dN/dS for Microsatellite stable (MSS), unstable (MSI) or PolE mutant tumors (POLE) from colorectal (CRC), stomach (STAD) and uterine carcinomas (UCEC). Dashed line represents Immune dN/dS one. Boxplots represent the median, 25th percentile, and 75th percentile, and whiskers correspond to 1.5 times the interquartile range. P-values were obtained from unpaired two-sided two-sample t-test. C) Immune dN/dS versus TMB for MSS escaped- and escaped+, MSI, and POLE tumors (n=491). Immune dN/dS versus TMB when restricting to D) all mutations (n=243) or E) only clonal mutations (n=175). F) Immune dN/dS versus TMB when restricting the analysis to patients with a minimum of 3 synonymous mutations in the immunopeptidome (n=39). P values and correlation coefficients were calculated using Pearson's correlation (two-sided t-test).

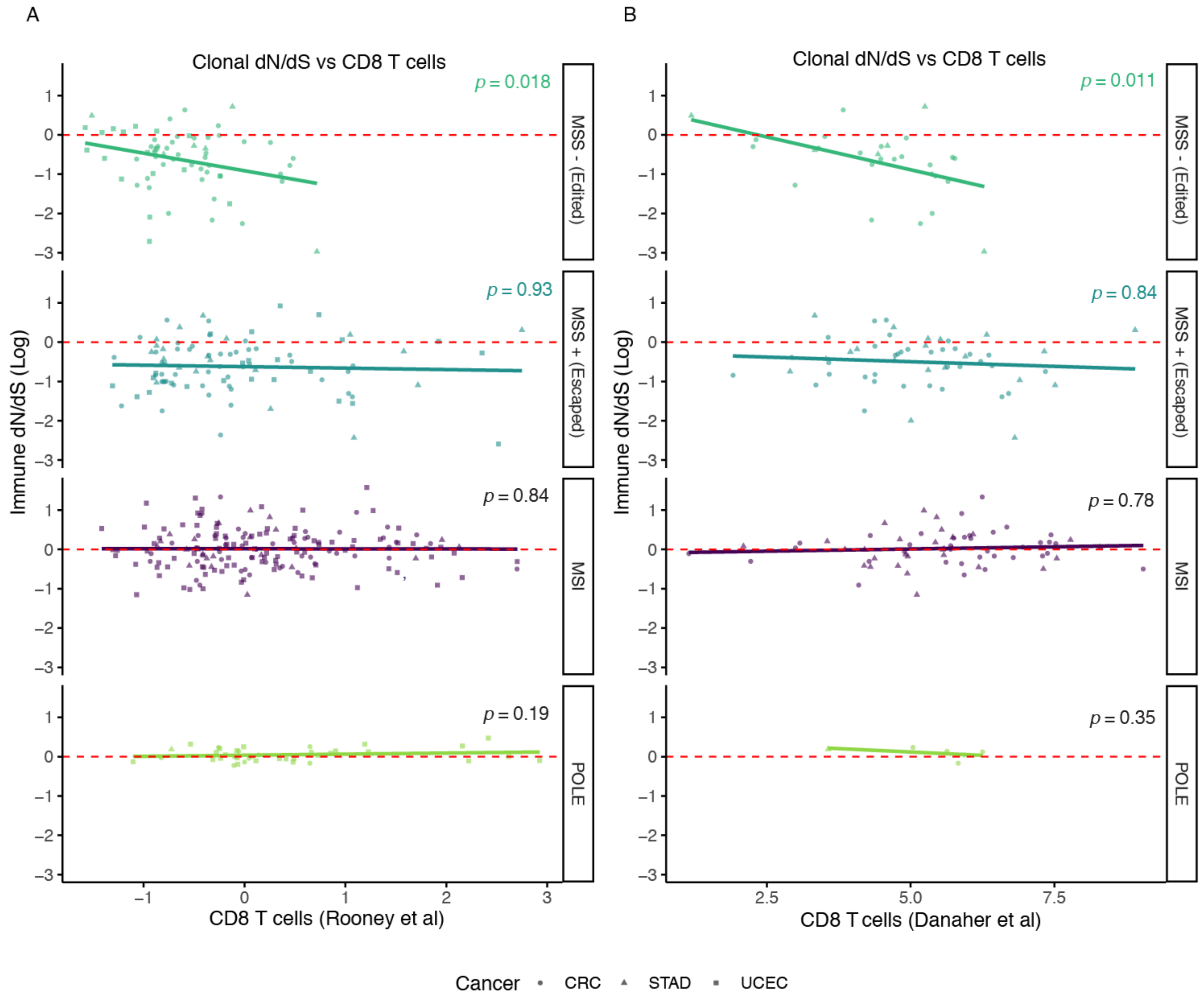

Supplementary Figure 10. Immune dN/dS using clonal mutations versus CD8 T cells infiltrate estimates from two sources A) Rooney et al 2015 and B) Danaher et al 2017 (n=393). P values and correlation coefficients were calculated using Pearson's correlation (two-sided t-test).

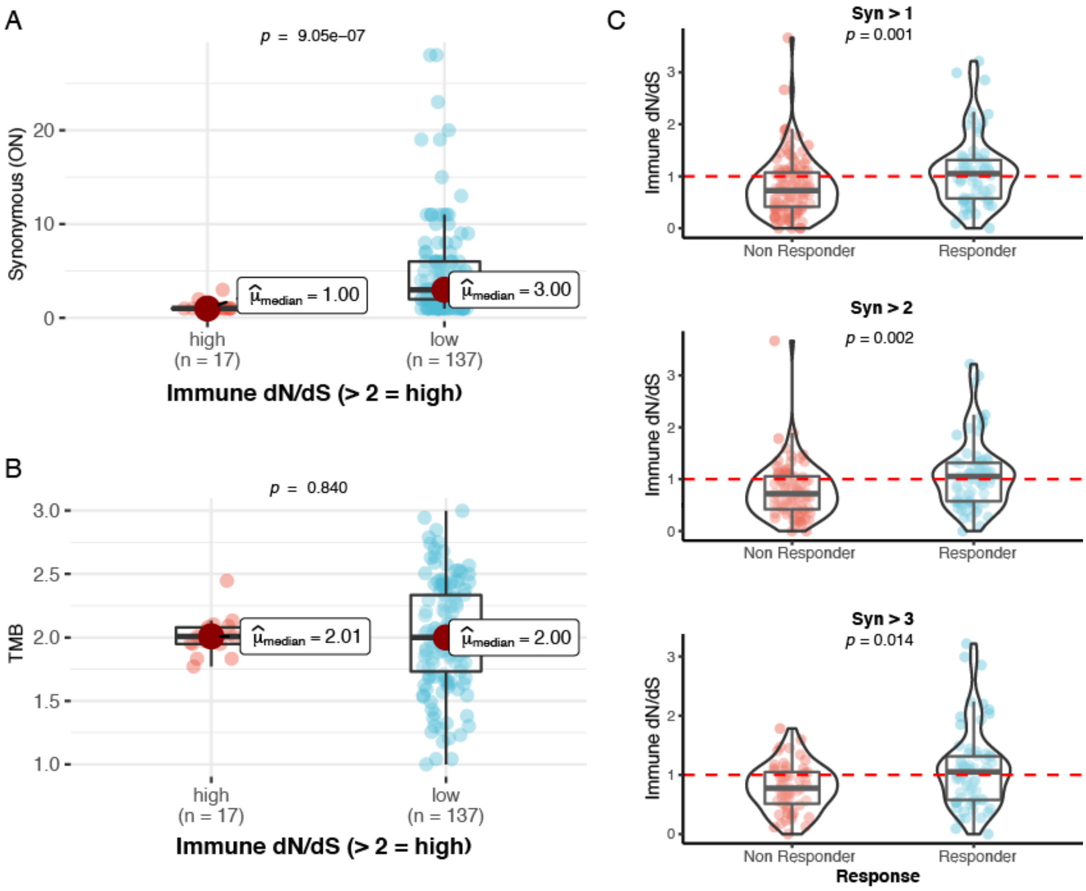

Supplementary Figure 11. A) Number of synonymous mutations in the immunopeptidome for high immune dN/dS (Immune dN/dS > 2) patients compared to the rest. B) Tumour mutation burden between high immune dN/dS patients and the rest. C) Immune dN/dS distribution when filtering non-responders with more than 1 (n=177), 2 (n=150) or 3 (n=124) synonymous mutations in the immunopeptidome. Boxplots represent the median, 25th percentile, and 75th percentile, and whiskers correspond to 1.5 times the interquartile range. P-values reported from a non-parametric two-sided Mann-Whitney U test after multiple test correction using Holm method.

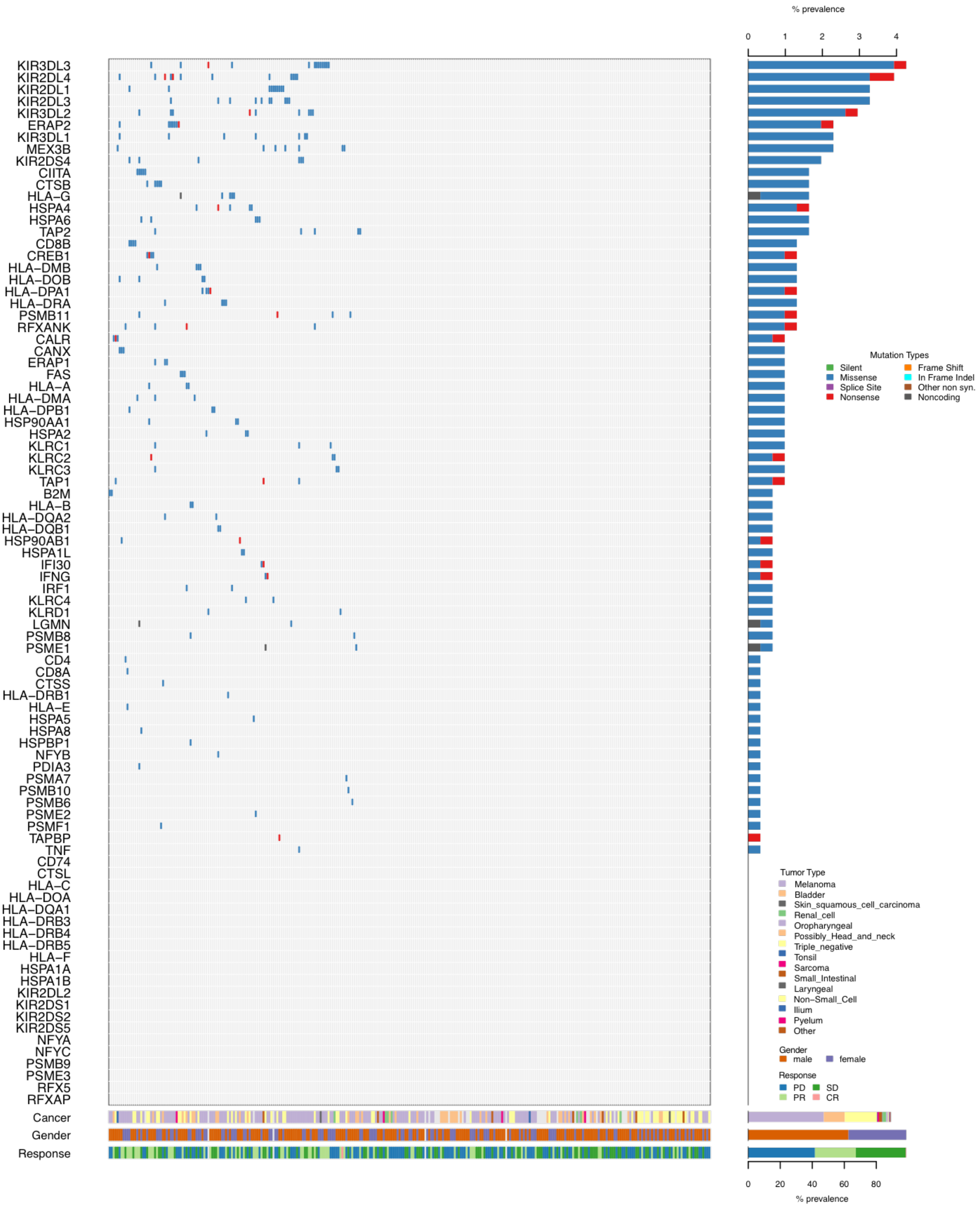

Supplementary Figure 12. Landscape of genetic alterations in escape genes for individuals from the Hartwig Medical Foundation cohort (n=308).

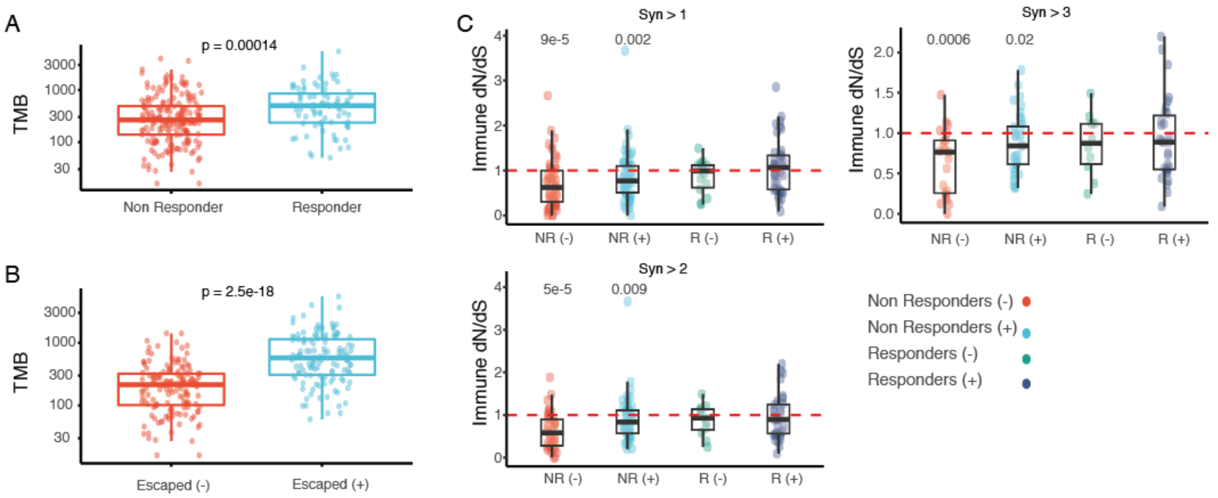

Supplementary Figure 13. A) TMB distribution for Non-Responders (n=193) and Responders (n=76). B) TMB distribution for escaped- (n=145) and escaped+(n=124) tumors. P-values reported from a non-parametric two-sided Mann-Whitney U test. C) Immune dN/dS distribution for 4 categories based on response and escape status for three synonymous mutations cut-off levels (min syn 2, n=163, min syn 3, n=126, min syn 4, n=97). Significant P-values reported using one-sided Wilcoxon test with mu=1. Boxplots represent the median, 25th percentile, and 75th percentile, and whiskers correspond to 1.5 times the interquartile range.

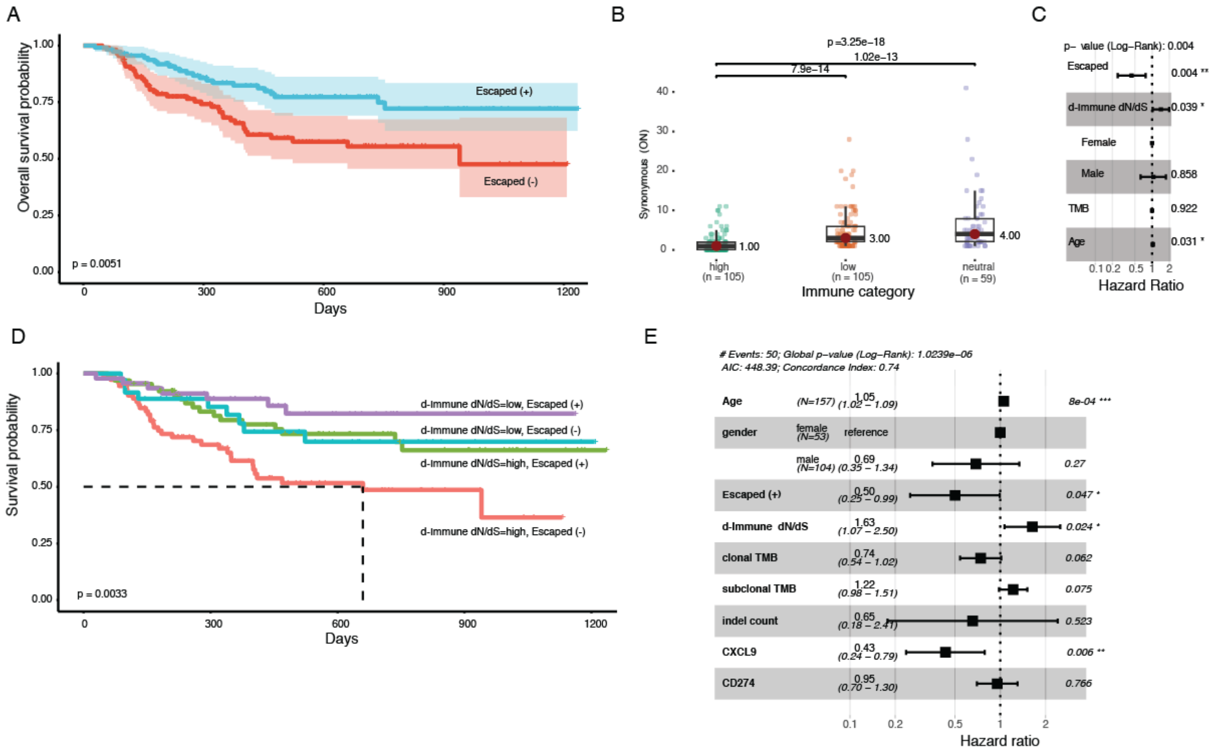

Supplementary Figure 14. A) Kaplan-Meier curves of overall survival between escaped+ versus escaped- in the HMF cohort (n=269). B) Number of synonymous mutations in the immunopeptidome for individuals classified as immune dN/dS low, neutral or high Boxplots represent the median, 25th percentile, and 75th percentile, and whiskers correspond to 1.5 times the interquartile range. P-values reported from a non-parametric two-sided Mann-Whitney U test after multiple test correction using holm method. C) Forest plot illustrates the HR (squares) and 95% CI (whiskers) for OS calculated using a multivariate cox proportional hazard regression model on selected features (n=221). D) Kaplan-Meier curves of overall survival between escaped+ versus escaped- further stratified by immune dN/dS distance to neutrality (n=221). E) Multivariate cox regression model including several response variables recently published in Litchfield et al 2022. Kaplan-Meier and forest plots display global log-rank test P-value adjusted using Benchamini-Hochberg multiple correction.

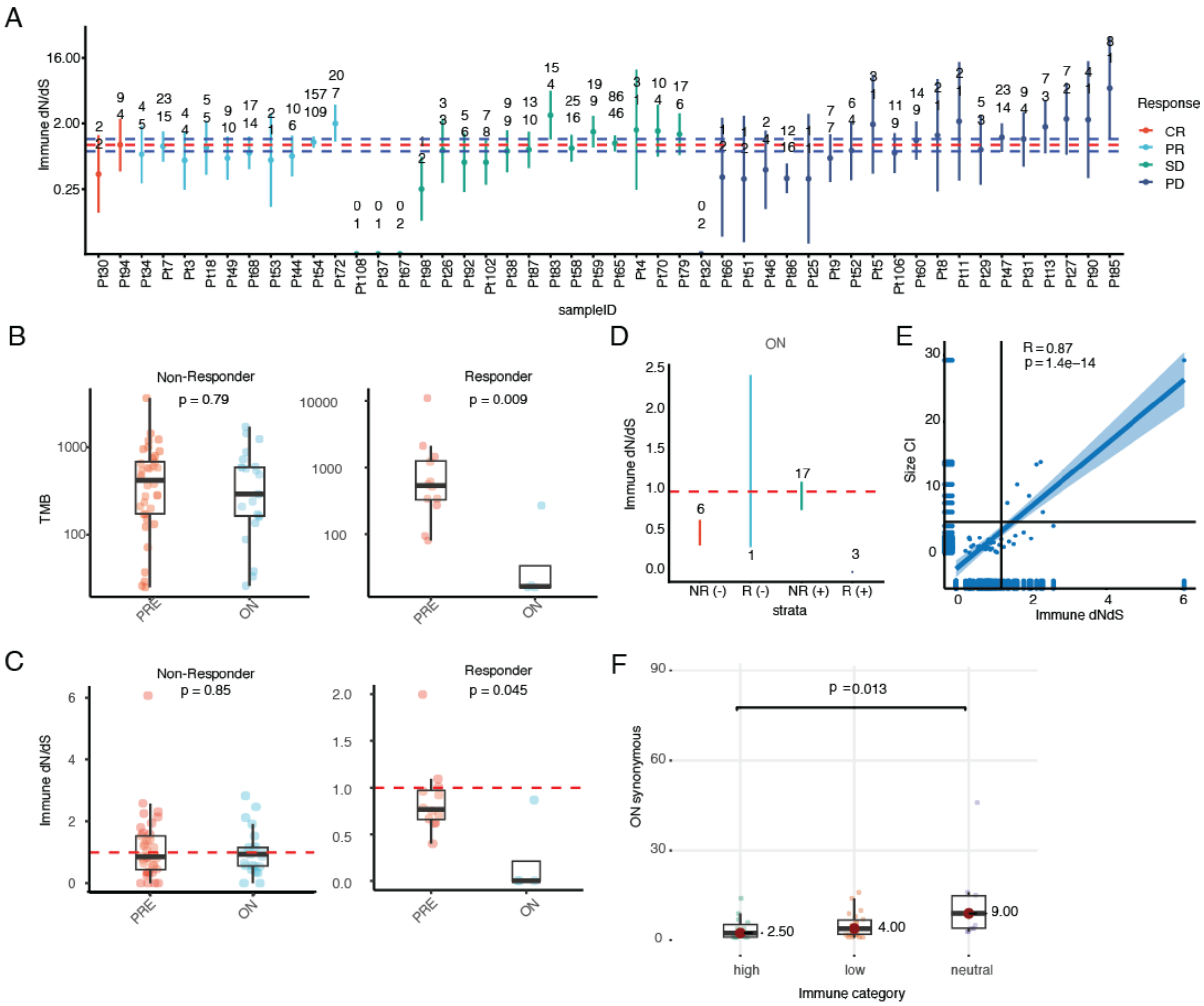

Supplementary Figure 15. A) Immune dN/dS values and their confidence intervals per patient. Each line is coloured by RECIST1.1 response category (CR-Complete response, PR-Partial response, SD-Stable disease and PD-Progressive disease). The upper and lower number are the number of nonsynonymous and synonymous mutations in the immunopeptidome respectively. B) TMB and C) immune dN/dS for a metastatic melanoma cohort sequenced before (PRE) and after (ON) immunotherapy for non-responders (n=36) and responders (n=12). D) Immune dN/dS during immunotherapy (ON) for groups classified based on their response and escape status. E) Scatter plot for immune dN/dS versus the size of the confidence interval.. The black lines are the selected cut-offs for analysing neutral (lower right quadrant) versus edited (low dNdS, lower left quadrant) patients. P values and correlation coefficients were calculated using Pearson's correlation (two-sided t-test). F) Number of synonymous mutations in the immunopeptidome for low, neutral and high immune dN/dS patients in the Riaz et al cohort. All error bars include the point estimate plus the 95% confidence interval calculated using SOPRANO package. Boxplots represent the median, 25th percentile, and 75th percentile, and whiskers correspond to 1.5 times the interquartile range. P-values reported from a non-parametric two-sided Mann-Whitney U test after multiple test correction using holm method.

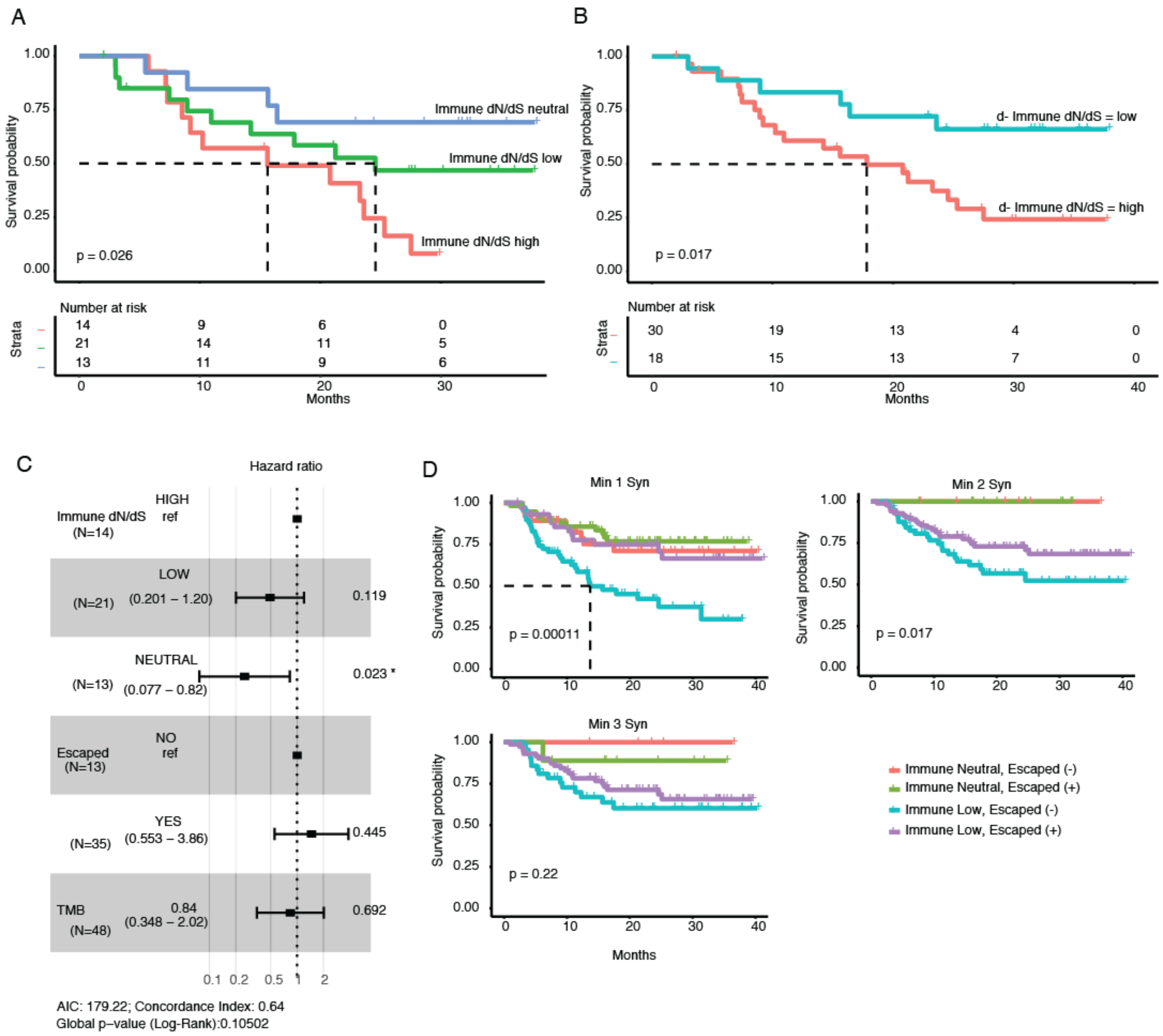

Supplementary Figure 16. A) Kaplan-Meier curves of overall survival for melanoma patients classified as immune dN/dS low (<0.82), neutral and high (>1.21). B) Kaplan-Meier curves of overall survival for melanoma patients classified based on their immune dN/dS distance to neutrality. C) Multivariate Cox-regression model using immune dN/dS categories, escape status and TMB for the Riaz cohort. D) Kaplan-Meier curves of overall survival for individuals from both cohorts (n=317), Hartwig medical foundation and Riaz, classified based on immune dN/dS and escape status when filtering for different minimum number of synonymous mutations in the immunopeptidome. Kaplan-Meier and forest plots display global log-rank test P-value adjusted using Benchamini-Hochberg multiple correction.

Supplementary Tables

Supplementary Table 1. TCGA tumour types analysed using SOPRANO.

Supplementary Table 2. Immune dN/dS results for each tumour type using SOPRANO. SOPRANO was run on different somatic callers, using different target HLA-alleles and different correction bias methods. In the manuscript we used the results from mutect, SSB192 on the protoHLA and the HLA-A0201 for the global analysis.

Supplementary Table 3. HUGO symbol of genes selected as "escape".

Suppementary Table 4. Patient specifc dN/dS estimates for TCGA individuals.

Suppementary Table 5. Escape status for TCGA individuals based on mutations on the list of escape genes.

Suppementary Table 6. dN/dS values for the list of escape genes using dndscv.
