# Supplementary material for: Immune selection determines tumor antigenicity and influences response to checkpoint inhibitors
Source: Nat Genet. 2023 Mar 9;55(3):451–60. doi: 10.1038/s41588-023-01313-1 (PMC10011129; doi:10.1038/s41588-023-01313-1)
Supplement: Supplementary file 2 — Reporting Summary [file 41588_2023_1313_MOESM2_ESM.pdf]

nature portfolio

Corresponding author(s): Liuis Zapata, Trevor Graham, Andrea Sottoriva

Last updated by author(s): Nov 22, 2021

Reporting Summary

Nature Portfolio wishes to improve the reproducibility of the work that we publish. This form provides structure for consistency and transparency in reporting. For further information on Nature Portfolio policies, see our Editorial Policies and the Editorial Policy Checklist.

Statistics

For all statistical analyses, confirm that the following items are present in the figure legend, table legend, main text, or Methods section.

n/a	Confirmed	
☐	☒	The exact sample size (*n*) for each experimental group/condition, given as a discrete number and unit of measurement
☒	☐	A statement on whether measurements were taken from distinct samples or whether the same sample was measured repeatedly
☐	☒	The statistical test(s) used AND whether they are one- or two-sided<br>*Only common tests should be described solely by name; describe more complex techniques in the Methods section.*
☒	☐	A description of all covariates tested
☐	☒	A description of any assumptions or corrections, such as tests of normality and adjustment for multiple comparisons
☐	☒	A full description of the statistical parameters including central tendency (e.g. means) or other basic estimates (e.g. regression coefficient) AND variation (e.g. standard deviation) or associated estimates of uncertainty (e.g. confidence intervals)
☐	☒	For null hypothesis testing, the test statistic (e.g. *F*, *t*, *r*) with confidence intervals, effect sizes, degrees of freedom and *P* value noted<br>*Give P values as exact values whenever suitable.*
☒	☐	For Bayesian analysis, information on the choice of priors and Markov chain Monte Carlo settings
☒	☐	For hierarchical and complex designs, identification of the appropriate level for tests and full reporting of outcomes
☐	☒	Estimates of effect sizes (e.g. Cohen's *d*, Pearson's *r*), indicating how they were calculated

Our web collection on statistics for biologists contains articles on many of the points above.

Software and code

Policy information about availability of computer code

Data collection	Data collection was performed using the R programming language (environment 3.63, 2020-02-29) and the TCGAbiolinks package. SOPRANO code is freely available at github.com/luisgls/SOPRANO. Code for simulator of stochastic branching process for immunoediting is available at github.com/luisgls/dNdSSimulator. Code for estimating positive selection in escape genes can be accessed from https://github.com/im3sanger/dndscv. Code for estimating driver, global and driver dN/dS can be obtained from github.com/luisgls/SSB_selection.
Data analysis	We used the R programming language (environment 3.63, 2020-02-29), and standard R packages available at repositories such as CRAN (2020-02-29) and Bioconductor 3.12. Bedtools 2.26 and R are needed for SOPRANO to run. The software produced for this publication is made available as described in the methods section of the paper. Tutorial to run SOPRANO is made available on github.com/luisgls/SOPRANO.samtools (v1.11) was used to convert fastq files. Yara Mapper (https://www.seqan.de/apps/yara.html) was used for read mapping. OptiType v1.3.3 was used to get HLA alleles. The references are based on the IMGT/HLA Release 3.14.0, July 2013. netMHCpan4.0 and 4.1 was used to predict MHC binding.

For manuscripts utilizing custom algorithms or software that are central to the research but not yet described in published literature, software must be made available to editors and reviewers. We strongly encourage code deposition in a community repository (e.g. GitHub). See the Nature Portfolio guidelines for submitting code & software for further information.

Data

Policy information about availability of data

All manuscripts must include a data availability statement. This statement should provide the following information, where applicable:

- Accession codes, unique identifiers, or web links for publicly available datasets
- A description of any restrictions on data availability
- For clinical datasets or third party data, please ensure that the statement adheres to our policy

TCGA data was obtained from GDC portal (https://portal.gdc.cancer.gov/) and processed as described previously. HLA Binding Mutation Rates (HBMR) values of selection in the immunopeptidome were obtained from the supplementary material in Van Den Eynden et al. Normalized scores for immune cell infiltration were obtained from Rooney et al, Danaher et al and Thorsson et al. Genes involved in the antigen presenting machinery were obtained from KEGG pathway hsa04612 (https://www.genome.jp/pathway/hsa04612). Assembled list of escape mechanisms for COAD, READ and STAD and UCEC was obtained from Lakatos et al. Somatic variant calls from 308 Hartwig Medical Foundation (HMF) samples were downloaded from the Hartwig Data Portal under license agreement DR-075 (https://database.hartwigmedicalfoundation.nl/). HMF Patient-level genome-wide germline and somatic data (raw BAM files and annotated variant call data) are considered privacy sensitive and available through an access-controlled mechanism. Somatic calls, clinical and HLA allele information from 68 metastatic individuals sequenced before and during immunotherapeutic treatment was obtained from the authors of Riaz et al and deposited in Zenodo (10.5281/zenodo.7546705). SOPRANO results for each tumor type and patient are available as supplementary tables. Analyzed data, code and R markdown files to reproduce raw figures have been made available in Zenodo (10.5281/zenodo.7416627).

Field-specific reporting

Please select the one below that is the best fit for your research. If you are not sure, read the appropriate sections before making your selection.

☒ Life sciences ☐ Behavioural & social sciences ☐ Ecological, evolutionary & environmental sciences

For a reference copy of the document with all sections, see nature.com/documents/nr-reporting-summary-flat.pdf

Life sciences study design

All studies must disclose on these points even when the disclosure is negative.

Sample size	All sample sizes are described in the manuscript. Table 1 contains information on sample size for data used. No sample size calculation was performed for patient selection as all samples available were used. A minimum number of mutations was used for estimating immune dN/dS as described in the manuscript.
Data exclusions	No data exclusions.
Replication	R Markdown files and data needed to replicate our results are deposited in synapse.
Randomization	We randomize genes as escape using same gene sample number to demonstrate the effect of immune selection in escape genes. This analysis involving randomization of samples is described in the manuscript
Blinding	n/a. Data collection involved selecting all samples available from three cohorts.

Reporting for specific materials, systems and methods

We require information from authors about some types of materials, experimental systems and methods used in many studies. Here, indicate whether each material, system or method listed is relevant to your study. If you are not sure if a list item applies to your research, read the appropriate section before selecting a response.

Materials & experimental systems

n/a	Involved in the study	
☒	☐	Antibodies
☒	☐	Eukaryotic cell lines
☒	☐	Palaeontology and archaeology
☒	☐	Animals and other organisms
☒	☐	Human research participants
☒	☐	Clinical data
☒	☐	Dual use research of concern

Methods

n/a	Involved in the study	
☒	☐	ChIP-seq
☒	☐	Flow cytometry
☒	☐	MRI-based neuroimaging
